# Biogasification of methanol extract of lignite and its residue: A case study of Yima coalfield, China

**DOI:** 10.1371/journal.pone.0275842

**Published:** 2022-10-12

**Authors:** Jianmin Liu, Hengxing Ren, Yi Jin, Huan He, Linyong Chen, Guofu Li, Baoyu Wang

**Affiliations:** 1 School of Resources & Environment, Henan Polytechnic University, Jiaozuo, P R China; 2 Collaborative Innovation Center of Coalbed Methane and Shale Gas for Central Plains Economic Region, Jiaozuo, P R China; 3 State Key Laboratory of Coal and CBM Co-mining, Jincheng, P R China; 4 School of Chemical Engineering and Technology, China University of Mining and Technology, Xuzhou, China; Universiti Teknologi Petronas, MALAYSIA

## Abstract

To investigate the biogas generation characteristics of the organic matter in lignite, methanol extraction was conducted to obtain the soluble fraction and the residual of lignite, which were subsequently taken as the sole carbon source for biogas production by a methanogenic consortium. Afterward, the composition of compounds before and after the fermentation was characterized by UV-Vis, GC-MS, and HPLC-MS analysis. The results indicated that the methanogenic microorganisms could produce H_2_ and CO_2_ without accumulating CH_4_ by utilizing the extract, and the methane production of the residue was 18% larger than that of raw lignite, reaching 1.03 mmol/g. Moreover, the organic compounds in the methanol extract were degraded and their molecular weight was reduced. Compounds such as 1, 6-dimethyl-4-(2-methylethyl) naphthalene, 7-butyl-1-hexylnaphthalene, simonellite, and retene were completely degraded by microorganisms. In addition, both aromatic and non-aromatic metabolites produced in the biodegradation were detected, some of which may have a negative effect on the methanogenesis process. These results revealed the complexity of the interaction between coal and organism from another point of view.

## Introduction

Coal-bed methane (CBM), a new energy currently being promoted and developed in China, is a clean energy of high quality. Many reports have indicated that nearly 20% of the methane gas in the developed CBM resources is produced by microorganisms [1]. This has drawn the attention of many researchers worldwide to the promotion of CBM production by microorganisms and proposed a mechanism for the bioconversion of coal to methane [2]. Biogas obtained from in-lab gas production simulation experiments typically consists of methane and unconverted hydrogen and carbon dioxide [3–5]. Although the methane concentration in the biogas is lower than that in the original coal seam, the presence of carbon dioxide allows the syngas to be further produced by other means such as methane dry reforming, providing a new way for the clean and efficient utilization of coal [6, 7].

Biogasification of coal is a process in which methane is produced by anaerobic degradation of organic matter in coal by methanogenic bacteria. During this process, soluble organic matter is released from coal and continuously degraded by microorganisms to form precursor substances such as acetic acid, carbon dioxide, hydrogen, and C1 compounds such as methanol, which are finally converted into methane [8–10]. Heteroatoms such as nitrogen, oxygen, and sulfur in coal macromolecules are considered to be the active sites of biodegradation [11–15]. The soluble oxygen-containing organics are readily released from coal and come into contact with microorganisms, however, their effects on each stage of the biogasification process are unknown.

Methanol, a relatively highly polar, readily available organic solvent, has enriched oxygen-containing compounds in coal [16]. In addition, the methanol extract of coal usually contains alkanes, aromatics and other heteroatomic compounds, which are involved in the biodegradation of coal [17–21]. Although aromatic compounds seem more resistant, they can still be degraded under anoxic conditions through the cleavage of aromatic rings [22, 23]. Solvent extraction also causes changes in coal organic composition and pore structure, which also have implications for microbial-coal interactions [24, 25]. Therefore, the research on the biodegradation of methanol extracts of coal and its residue is beneficial for exploring the interaction mechanism between microorganisms and coal.

In this research, methanol was employed as an organic solvent for the Soxhlet extraction to obtain the organic matter of Yima lignite. And microorganisms with good anaerobic gas production effect on lignite, pre-stored in the laboratory, were used as bacterial sources. Afterward, extracts and residue were utilized as substrates to conduct gas production simulation experiments. Finally, various analytical methods were combined to analyze the gas production of extracts and residue, as well as the composition and content of organic matter in the gas production process. This research provided an experimental basis for the subsequent analysis and degradation mechanism of biogas-generating active organic components in coal.

## Materials and methods

### Lignite methanol extraction and GC-MS analysis

Lignite was collected from the No. 2–3 coal seams of Qianqiu Mine in Yima field in Henan province, with a buried depth of 798.5 m and a coal thickness of 6.82 m. The sedimentary age of the lignite sample with *Ro* = 0.48% was the Middle Jurassic. After sampling on site, coal was stored in a nitrogen-filled sealed tank. Before conducting the experiments, the oxide layer was removed and pulverized to below 120 meshes. After being dried at 70°C to a constant weight, it was stored in a sample bag and named YM.

The methanol extraction process of coal was as follows: (1) 50 g of lignite was weighed, and 250 mL methanol was employed as the extraction solvent to perform Soxhlet extraction at 68°C for 80 h [17]; (2) after the extraction was completed, the extract was concentrated at 45°C using a rotary evaporator. Then, the concentrated extract was made up to 100 mL with methanol and recorded as M1; (3) the residual coal was dried to a constant weight at 70°C and stored in the sample bag, recorded as M2. The calculation formula for methanol extraction rate was as follows:

$$P=\left(m_{0}-m_{1}\right)/m_{0}\times 100\%$$ (1)

where *P* is the extraction rate; *m*_*0*_ is the mass of raw coal, and *m*_*1*_ is the mass of residual coal. The methanol extraction rate of Yima lignite was 3.12%.

After diluting M1 10 times with methanol, the organic components of the extracts were analyzed by gas chromatography-mass spectrometry (GC-MS, Agilent 7890A-5795C). The column was VF-WAXms (30 m×250 μm×0.25 μm), the post-operation temperature was 280°C which was maintained for 5 min, and no split injection was applied. The inlet temperature was 250°C, the injection volume was 0.8 μL, the purging rate was 15 mL/min, and the purging time was 0.2 min. Also, the carrier gas was helium with high purity, the column flow rate was 1.0 mL/min, and the initial temperature was 60°C which was kept for 2 min. Then the temperature was raised to 250°C at a rate of 10°C/min and was maintained for 20 min. The MS was operated in the electron impact mode, with an ionization energy of 70 eV. The mass spectrometric identification was performed using the mass spectral database NIST2008.

### Biogas production experiment of methanol extract and residue from lignite

The biogas production experiment consisted of four experimental groups: M1, M2, YM, and CK. All groups were set up in triplicate. The substrate of each experimental group was M1 2 mL, M2 2 g, YM 2 g, and methanol 2 mL, respectively. The components of the medium used in the experiment were as follows: K_2_HPO_4_ (2.9 g), KH_2_PO_4_ (1.5 g), NH_4_Cl (1.8 g), MgCl_2_ (0.4 g), yeast extract (0.2 g), L-cysteine hydrochloride (0.5 g), deionized water (1000 mL).

Microorganisms with a good anaerobic gas production effect on lignite, pre-stored in the laboratory, were employed as bacterial sources. The bacterial group composition at the level of phylum predominantly included: *Firmicutes* (27%); *WWE1*(25%); *Bacteroidetes* (21%), *Synergistetes* (13%), *Proteobacteria* (5%), and *Chloroflexi* (1%), and *Archaea* which mainly belonged to the *Euryarchaeota*. The total number of bacteria per ml was 1.3 × 10^7^, and the inoculation amount was 4%. The experimental period was 180 d.

Gas chromatography (GC, Agilent 7890) was employed to analyze the gas composition in the process of biogas production, with a Carbonplot column (60 m×320 μm×1.5 μm), TCD detector, and gas-tight injection needle. The injection volume was 0.5 mL. The inlet temperature, column temperature, and the detector temperature were 150°C, 30°C, and 200°C, respectively.

### Organic composition analysis of extract and residue gas production system

The fermentation broth was initially aspirated from the anaerobic bottle with a sterile sampler. Then it was collected through a 0.22 μm microporous filter membrane. A dual-beam ultraviolet-visible light spectrophotometer (UV-Vis, Unico, UV4802) was employed to perform spectral scanning. The scanning range was 190–400 nm with an interval of 1 nm. The deionized water was used as a blank to qualitatively analyze the composition of organic matter in the fermentation broth.

Quantitative analysis of polar organic components of the fermentation broth was also carried out by liquid chromatography-mass spectrometry (HPLC-MS, Agilent 1290 6530 QTOF equipped with electrospray ionization source) equipped with an Agilent Zorbax C8 (1.8 μm×4.6 mm×50 mm). The mobile phase was methanol and 0.1% formic acid with a 0.5 mL/min flow rate. The column temperature was 25°C, and the injection volume was 10 μL. The mass spectrometry acquisition mode was positive ion mode, and the fragmentor voltage and the capillary voltage were 130 V and 3500 V, respectively. N_2_ was used as collision gas and dryer gas. The mass-to-charge ratio scanning range was 50–450 m/z.

The non-polar organic matter in the fermentation broth was initially enriched with a solid-phase extraction column (Agilent Bond Elut C18, 500 mg, 120 μm, 6 mL) through the following steps: (1) 6 mL of methanol and 6 mL of deionized water were added to the extraction column sequentially to activate it. (2) 10 mL of the sample filtered with a 0.22 μm microporous membrane was added and passed the column at a flow rate of about 2 mL/min. (3) The column was rinsed with 10 mL of deionized water initially, and then the column was blow-dried with nitrogen for 10 min. (4) The organic matter was eluted with 2 mL of methanol. Then the eluent was concentrated to a volume of 1 mL with nitrogen at 45°C. After the enrichment, the sample was analyzed using the GC-MS method described in section 2.1.

## Results and discussion

### The organic composition of methanol extract of lignite

**Fig 1:**
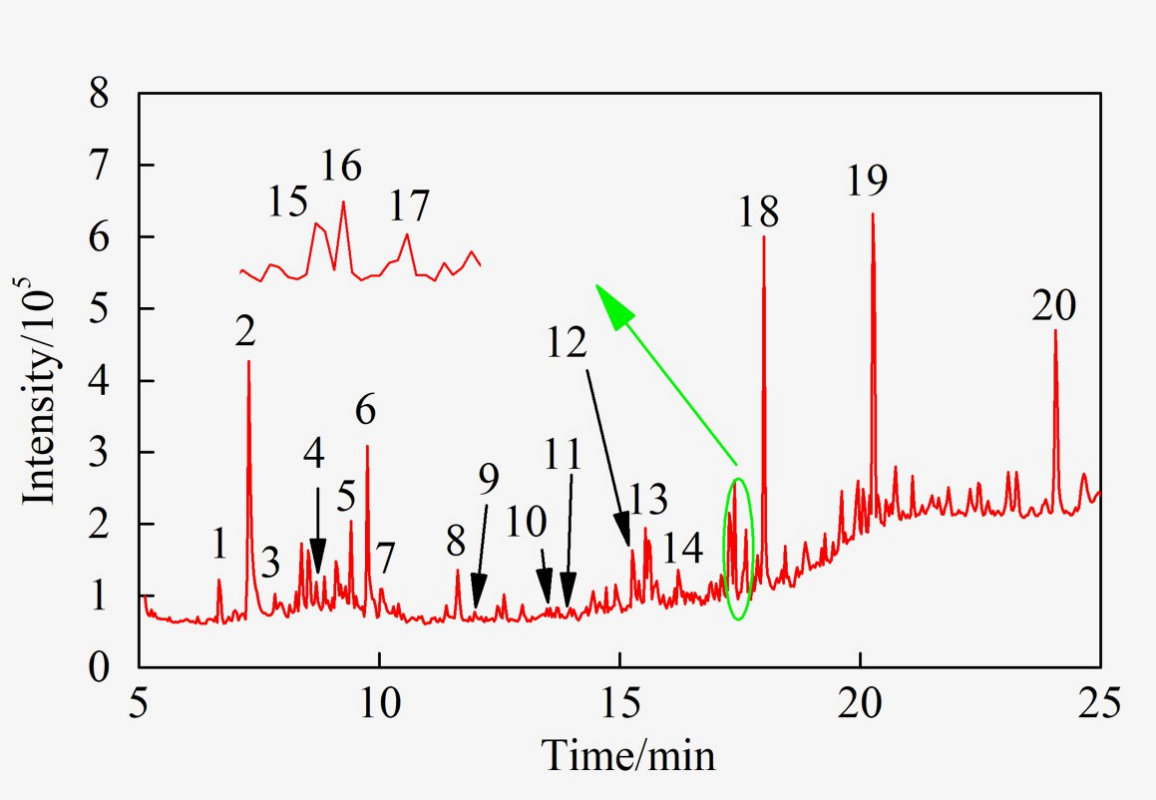
TIC of GC-MS of methanol extract.

The GC-MS total ion current chromatogram (TIC) of the extract is shown in Fig 1. The library search was performed on the chromatographic peaks. The 20 compounds with a matching degree greater than 60 are shown in Table 1.

**Table 1:** Identified molecular compounds in methanol extract.

NO.	Retention time/min	Composition	MW	Name	Prob.
1	6.697	C_4_H_6_O_4_	118	Dimethyl oxalate	80
2	7.314	C_2_H_4_O_2_	60	Acetic acid	90
3	7.828	C_2_H_5_BrO	124	2-bromoethanol	77
4	8.684	C_7_H_14_O_3_	146	Pentanoic acid, 2-hydroxy-4-methyl-, methyl ester	73
5	9.438	C_11_H_18_	150	Trans-1,2,3,4,4a,5,8,8a-octahydro-4a-methylnaphthalene	80
6	9.78	C_14_H_22_O	206	3,5-bis(1,1-dimethylethyl)-phenol	87
7	10.054	C_8_H_14_	110	1,2-dimethylcyclohexene	84
8	11.63	C_15_H_22_	202	1-methyl-4-(1,2,2-trimethylcyclopentyl)benzene	98
9	12.007	C_4_H_8_O	72	Trans-2,3-dimethylethylene oxide	79
10	13.72	C_4_H_8_O	72	Cis-2,3-dimethylethylene oxide	73
11	13.96	C_12_H_13_NO	187	1-(1,3-dimethyl-1H-indol-2-yl) ethanone	81
12	15.296	C_13_H_9_F_2_NO	233	N-(4-fluorophenyl)-3-fluorobenzamide	73
13	15.57	C_17_H_34_O_2_	270	Methyl hexadecanoate	95
14	16.255	C_14_H_13_N_3_O_2_	255	2-(3,4-dimethoxyphenyl)-1H-imidazo[4,5-c]pyridine	76
15	17.146	C_10_H_13_NO_3_	227	2- [2-(4-nitrophenoxy)ethoxy]ethanol	72
16	17.317	C_14_H_18_	186	(2-ethyl-3,3-dimethyl-cycloprop-1-allyl)benzene	79
17	17.626	C_15_H_18_	198	1,6-dimethyl-4-(2-methylethyl)naphthalene	77
18	18.002	C_20_H_30_	270	Dehydroabietane	74
19	18.893	C_20_H_28_	268	7-butyl-1-hexylnaphthalene	73
20	20.263	C_19_H_24_	252	Simonellite	71
21	24.1	C_18_H_18_	234	Retene	99

Alcohols, esters, carboxylic acids, amides, phenols, aromatic hydrocarbons, and heterocyclic compounds were mostly detected in the methanol extract. More than half of these compounds contained oxygen atoms, indicating that methanol extraction had an enrichment effect on oxygen-containing compounds in coal [16]. The oxygen element in the extract primarily existed in the form of hydroxyl, carbonyl, ester and amide groups, and the nitrogen element chiefly existed in the form of the heterocycle. The presence of nitrogen and oxygen heteroatoms provided potential sites for biodegradation. The aromatic hydrocarbons in the extract were principally alkyl substituents of naphthalene and phenanthrene, of which 7-butyl-1-hexylnaphthalene showed the highest abundance.

### Analysis of biogas production composition of lignite methanol extract and residual coal

**Fig 2:**
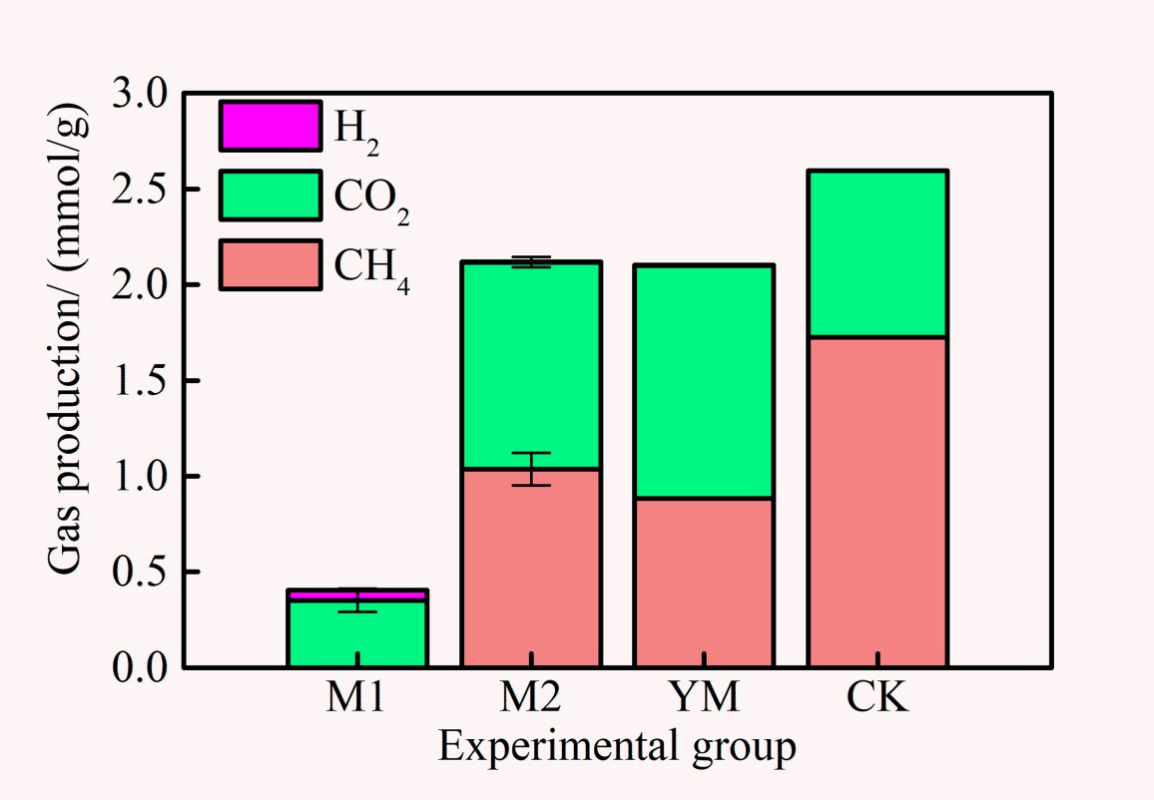
Biogenic methane production of each experimental group.

The results of biogas production in each experimental group are illustrated in Fig 2. Among the experimental groups, the M1 group had the largest H_2_ production (0.72%, the cumulative H_2_ production was 0.05 mmol/g). Also, all groups produced CH_4_ (methane content of CK>M2>YM), except the M1 group. The CK group had a large amount of CH_4_, CO_2_, and a small amount of H_2_, which indicated that the bacteria employed could directly use methanol as a substrate to produce methane. Furmann et al. [14] found that a small amount of CH_4_ could be detected after the anaerobic degradation of the methanol extract of highly volatile bituminous coal. However, only H_2_ and CO_2_ were detected in the M1 group, which contained organic matter extracted from coal in addition to methanol. It was thought that the extracted organic matter was the substrate of the fermentation bacteria and hydrogen-producing acetogens in the flora. The substrate promoted the production of more H_2_, but it did not play a corresponding role in the methanogenic process in the system.

Methane was produced by acetoclastic, methylotrophic, and hydrogenotrophic methanogens [2]. The largest abundance of methanogens in the original flora in this study was *Methanoculleus* [3], the most reported methanogenic microorganism in the literature. It can produce methane using small molecules such as H_2_/CO_2_ and formate. Theoretically, the M1 group should be able to produce methane. However, based on the current results, it was likely that the organic matter in the extract or the intermediate metabolites produced by the organic matter had a negative impact on the metabolic process of the methanogens. The specific reason still needed to be further elaborated on.

In contrast, after methanol extraction, the methane production of the M2 group reached 1.03 mmol/g and increased 18% compared with the YM group. It was speculated that methanol extraction increased the contact area between coal and microorganisms [26, 27].

### UV-vis analysis of organic matter in the biogas production system of lignite methanol extract and residual coal

**Fig 3:**
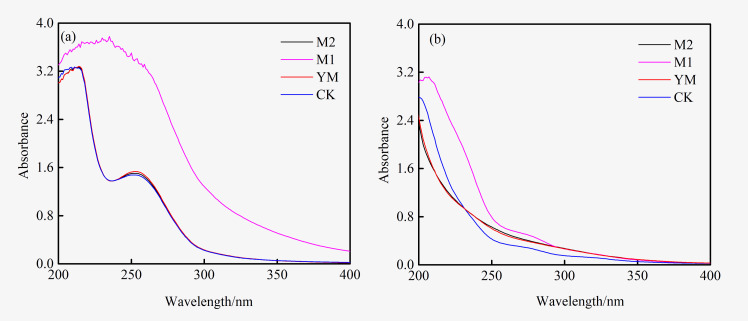
UV-vis analysis of fermentation liquid before and after biogenic methane production.

The results of the UV-vis analysis of fermentation broth before and after biogas production are exhibited in Fig 3. Before the gas production, the M1 group fermentation broth showed a strong continuous absorption (peak at 225 nm-275 nm), which was generated by the conjugation of the chromophore’s double bond with the benzene ring in the system [28]. Combined with the GC-MS analysis results of the extract, the absorption peak primarily originated from aromatic ketones and esters. The M2, YM, and CK groups had shoulder peaks at 220 nm and 254 nm, predominantly generated by the absorption of organic matter in the medium. After biogas generation, the absorption of the M1 group at 225–275 nm was significantly weakened. This result suggested that organic compounds such as aromatic ketones and esters in the extract were degraded. The M1 group had shoulder peaks near 220 nm and 280 nm, primarily from aromatic compounds [29]. However, the M2 group and YM group had no shoulder peaks in this wavelength range, which indicated that the products of the M1 group contained more aromatic compounds.

**Fig 4:**
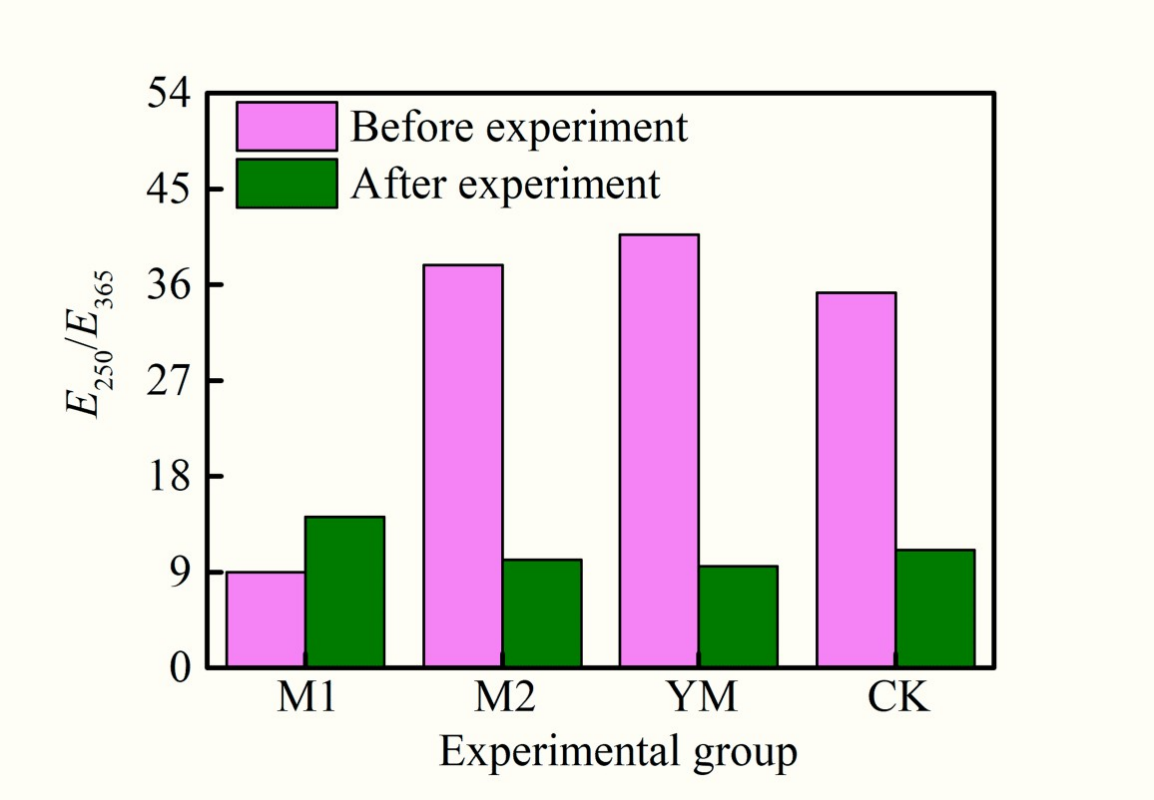
The changes of E_250_/E_365_ before and after biogenic methane production.

The ratio of the absorbance of the fermentation broth at 250 nm and 365 nm (E_250_/E_365_) was applied to characterize the molecular weight of soluble organic matter. The larger the ratio, the smaller the molecular weight of the organic matter is [29, 30]. The changes of E_250_/E_365_ before and after gas production in each experimental group are displayed in Fig 4. It can be observed that the fermentation broth of the M1 group contained a large amount of macromolecular organic matter before biogas production, and its E_250_/E_365_ was substantially smaller than that of other experimental groups. E_250_/E_365_ of the M1 group increased slightly after gas production, demonstrating that some organic substances were biodegraded and the molecular weight became smaller. While, E_250_/E_365_ of the M2 and YM groups decreased after gas production, which may be due to the release of macromolecular substances in the coal under the biological action [29].

### Composition and change of organic matter in the biogas production system of lignite methanol extract and residual coal

**Fig 5:**
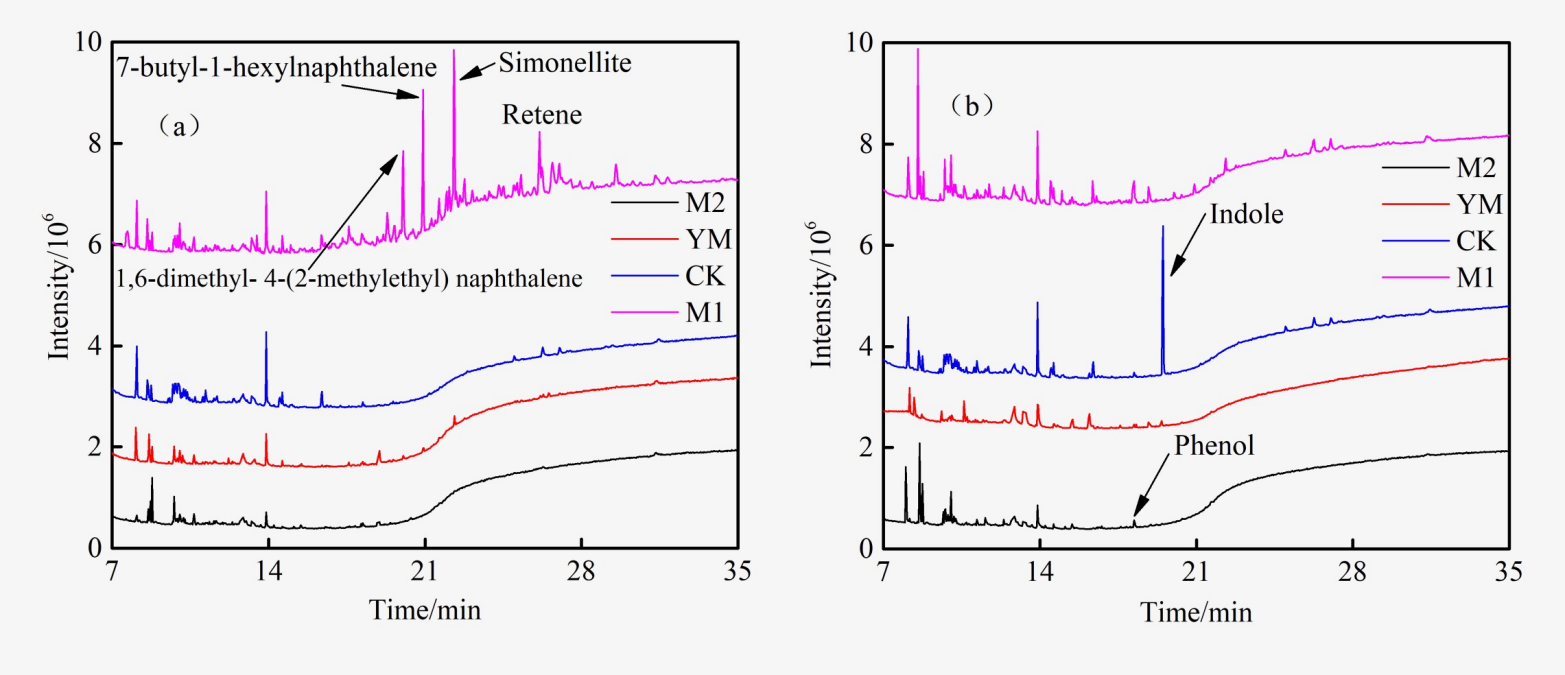
TIC of GC-MS of experimental groups before (a) and after (b) biogenic methane production.

The GC-MS total ion current chromatogram of the fermentation broth of each experimental group before and after gas production is shown in Fig 5. Before gas production, eight compounds were detected in the samples of the M1 group. They included trans-1,2,3,4,4a,5,8,8a-octahydro-4a-methylnaphthalene (No.5 in Table 1), 3,5-bis (1,1-dimethylethyl)-phenol (No. 6 in Table 1), 1-(1,3-dimethyl-1H-indol-2-yl) ethanone (No. 11 in Table 1), methyl hexadecanoate (No. 13 in Table 1), 1,6-dimethyl-4-(2-methylethyl) naphthalene (No. 17 in Table 1), 7-butyl-1-hexyl Naphthalene (No. 19 in Table 1), simonellite (No. 20 in Table 1), and retene (No. 21 in Table 1). No compounds were detected in the remaining experimental groups. After gas generation, the height of the main peaks in the chromatograms of the M1 group samples decreased significantly. The peaks of 1,6-dimethyl- 4-(2-methylethyl) naphthalene (No. 17 in Table 1), 7-butyl-1-hexylnaphthalene (No. 19 in Table 1), simonellite (No. 20 in Table 1), and retene (No. 21 in Table 1) disappeared. This result demonstrated that these compounds could be degraded and utilized by the flora. Different studies have reported that microorganisms of the *Firmicutes* and *Proteobacteria* can degrade polycyclic aromatic hydrocarbons [31, 32], and the microorganisms of the *Bacteroidetes* can degrade phenols [33–35]. These microorganisms in the inoculum might be involved in the degradation of organic matter in the extract.

A large amount of indole which was a metabolite produced by the use of methanol by the flora was detected in the fermentation broth after gas production in the CK group (Fig 5B). However, indole was not detected in the M1 group. It was speculated that some compounds might inhibit the production of indole.

Phenolic organic compounds could be connected with the degradation of lignin [36]. Phenol was found in the M1, M2, and YM groups after the experiment (Fig 5B), indicating the decomposition of polymers in lignite [37]. Moreover, these authors identified over 100 compounds from kerogen due to the decomposition of polymers, covering the compounds obtained in this study. Nevertheless, no more metabolites except phenol were detected in all groups. The possible reason was that the produced metabolites were polar and difficult to be detected using the GC-MS method. Therefore, HPLC-MS was employed for further analysis of the fermentation broth.

**Fig 6:**
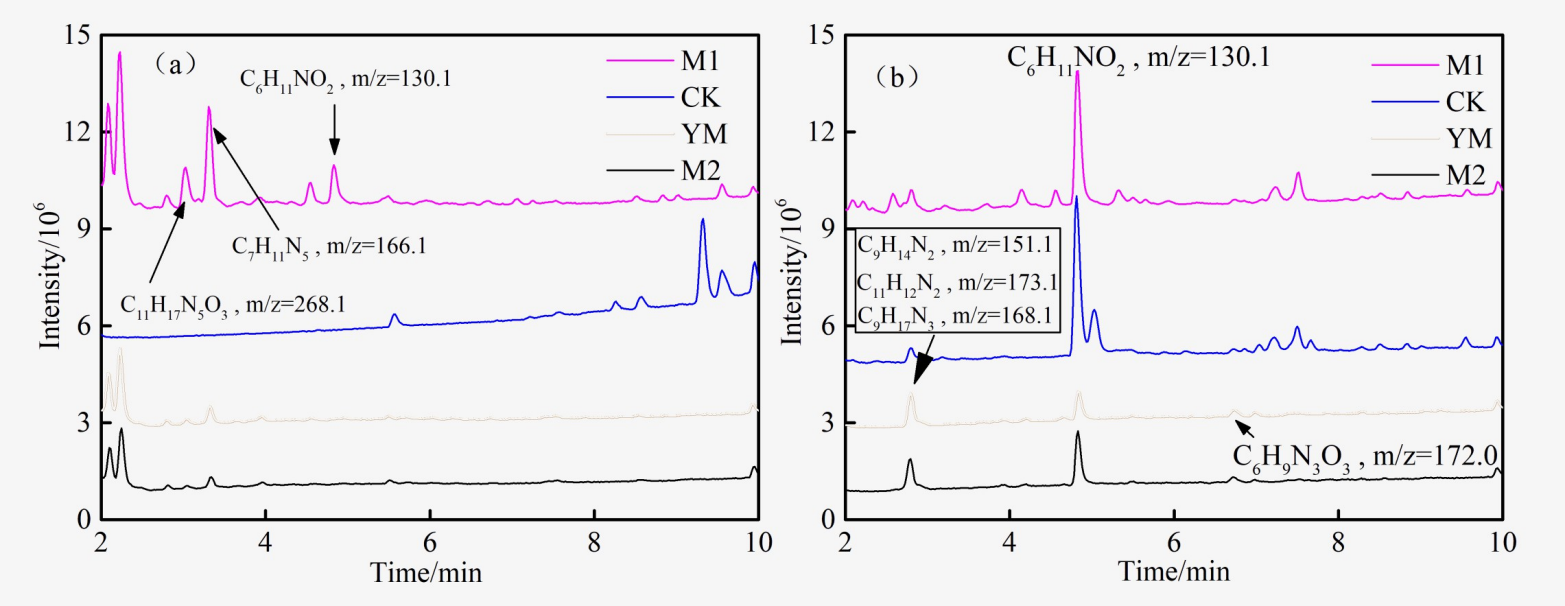
TIC of HPLC-MS of experimental groups before (a) and after (b) biogenic methane production.

The HPLC-MS total ion current chromatogram of the fermentation broth of each experimental group was exhibited in Fig 6. For samples of the M1 group after the experiment, the peaks of C_11_H_17_N_5_O_3_ (at 3.0 min), C_7_H_11_N_5_ (at 3.3 min), and C_13_H_11_NO_3_ (at 3.9 min) disappeared (Fig 6A), indicating that these compounds were degraded by the flora. Moreover, the abundances of C_9_H_14_N_2_ (at 2.8 min), C_11_H_12_N_2_ (at 2.8 min), C_9_H_17_N_3_ (at 2.8 min), C_6_H_11_NO_2_ (at 4.8 min), and C_6_H_9_N_3_O_3_ (at 6.7 min) increased significantly after the experiment (Fig 6B), which proved that these compounds were produced during the degradation of the extract. At the same time, YM and M2 groups also showed similar changes. Except for C_6_H_11_NO_2_, the number of rings plus double bonds of other compounds was more than 4. Combined with the results of UV analysis (Fig 3B), these products may be aromatic compounds.

The degradation of the extract might lead to the rapid accumulation of some metabolites, which may have a negative effect on the methane production process. Especially, C_6_H_11_NO_2_ existed in the extract and accumulated significantly after biological action in all groups. In another study, the addition of excess pulverized coal resulted in the inhibition of methanogenesis and the accumulation of C_6_H_11_NO_2_, which also showed that C_6_H1_1_NO_2_ was involved in inhibiting the methanogenesis process [38]. Interestingly, it was not released from coal during the sterilization, which indicated that it was firmly bound to the macromolecular structure. Considering the potential effect of C_6_H_11_NO_2_ on the methanogenesis process, its existence in coal also provided insight into the complexity of the process of microbial action.

**Fig 7:**
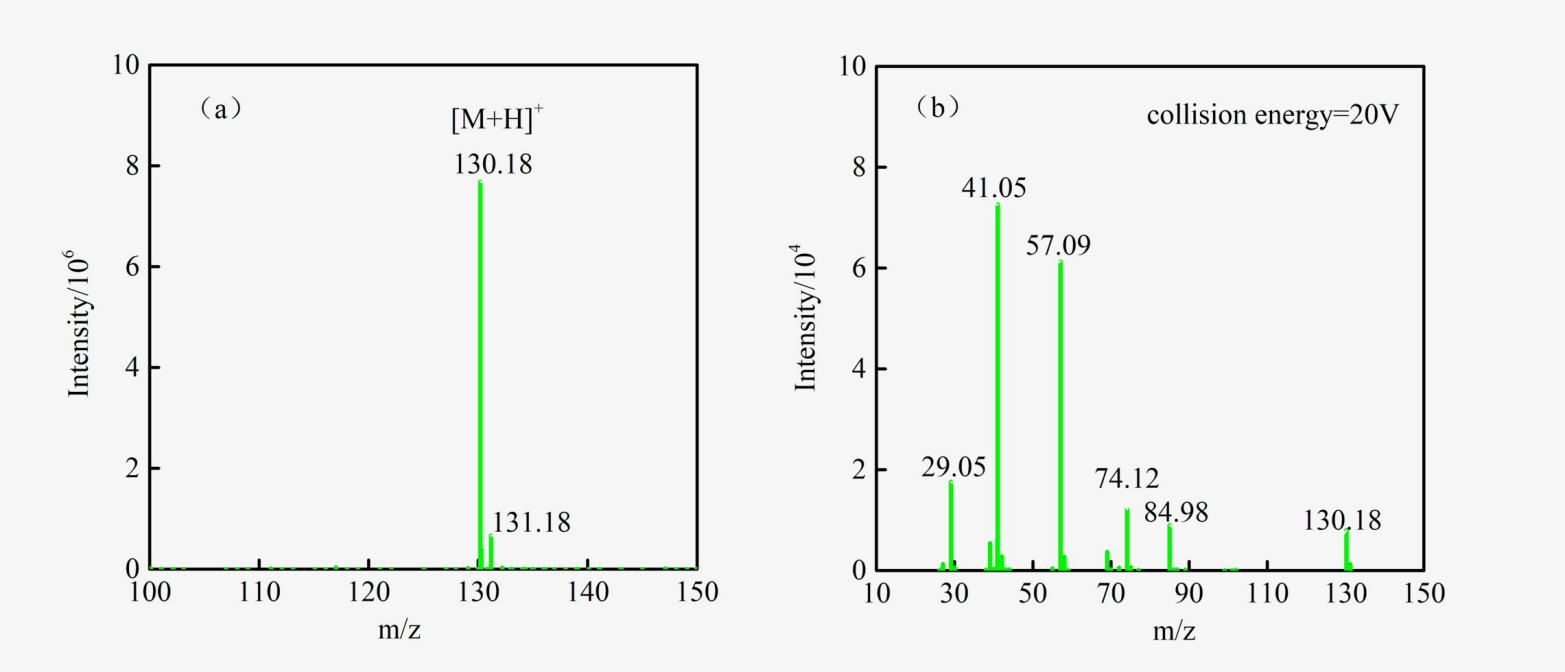
The MS(a) and MS/MS(b) spectrum of C_6_H_11_NO_2_.

The number of rings plus double bonds of C_6_H_11_NO_2_ is 2, indicating that it is not an aromatic compound. The MS and MS/MS spectra of C_6_H_11_NO_2_ are shown in Fig 7, and their specific structure needs to be further identified.

## Conclusions

Research on bioavailable organic matter in coal and its metabolites is an important part of elucidating the mechanism of coal biogas formation. In this study, many alcohols, esters, carboxylic acids, amides, phenols, aromatic hydrocarbons, and heterocyclic compounds were detected in the methanol extract of Yima lignite, with aromatic compounds showing the most considerable abundance. The methanol extract of Yima lignite was used by the microbial flora to produce H_2_ and CO_2_ without accumulation of CH_4_. Both Aromatic hydrocarbons and other oxygen-containing compounds in the extract were biodegraded, and their molecular weights decreased. Certain soluble organic compounds in the extract and metabolites of the biodegradation process may negatively affect the methanogenesis process, where the compound C_6_H_11_NO_2_ present in both the extract and the product may be the primary inhibitor. The methane production of residual coal increased by 18% compared with the raw coal. In addition, both aromatic and non-aromatic compounds were produced during the biodegradation process, and the appearance of phenol in the product indicated the depolymerization of lignin in coal. The current study demonstrated the complex role of soluble organic matter and its metabolites during the biogasificaition process.
